# Case report: Castleman’s disease in trachea

**DOI:** 10.1259/bjrcr.20160063

**Published:** 2017-01-25

**Authors:** Hong Xie, Shi Zhou, Chaonan Deng, Jun Jiao, Chao Fan, Yan Zhang

**Affiliations:** ^1^Department of Radiology, Gui Zhou Medical University, Guiyang, China; ^2^Department of Pathology, Gui Zhou Medical University, Guiyang, China; ^3^Departmant of surgery, Guizhou Medical University, Guiyang, China

## Abstract

Castleman’s disease (CD) in trachea is rarely reported to date. This report introduces a case of CD in trachea with emphasis on its radiological presentation onCT. A female patient was admitted at our emergency department with dyspnoea. Plain and enhanced CT revealed a mass with distinct border and marked enhancement. Tracheotomy was planned and it turned out to be CD of hyaline type on pathological examination. Two similar case reports on intratracheal CD from 1954 to 2015 were reviewed as we searched in PubMed using key words “endotracheal Castleman’s disease” or “Castleman’s disease in trachea” or “Castleman’s disease in tracheal”. This will be the third case report of CD in trachea in English literature. In this case report, the radiological appearance of CD on multiple imaging modes is reviewed. Lesions that should be taken into consideration in differential diagnosis are mentioned. The two main surgical methods for such lesions are briefly described.

## Case report

**Figure 1.**
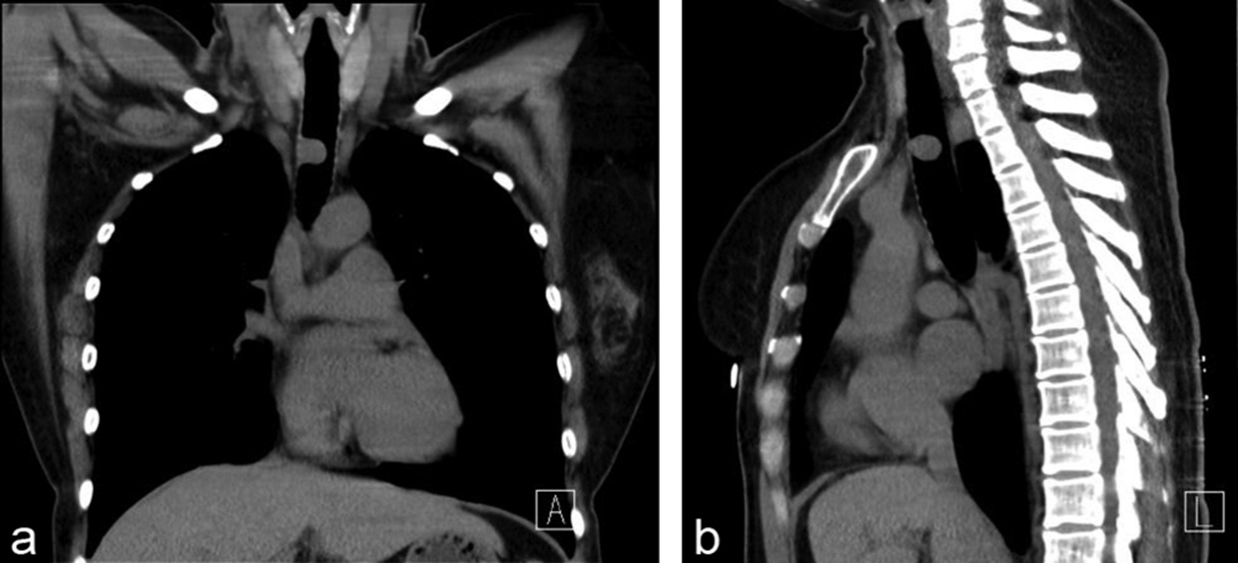
Plain CT (a, b) of the chest demonstrates a mass with soft-tissue density, about 2 × 1 × 1 cm^3^, on the lateral wall of the trachea. (a) Coronal plane. (b) Sagittal plane.

**Figure 2.**
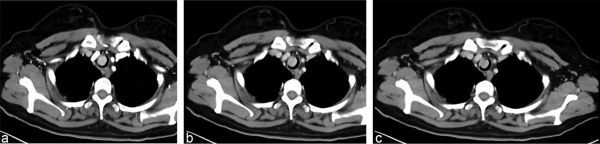
Contrast-enhanced CT of the chest (a–c) 30, 60 and 120 s after injection. Density of the lesion: (a) 122 HU; (b) 103 HU; (c) 85 HU.

**Figure 3.**
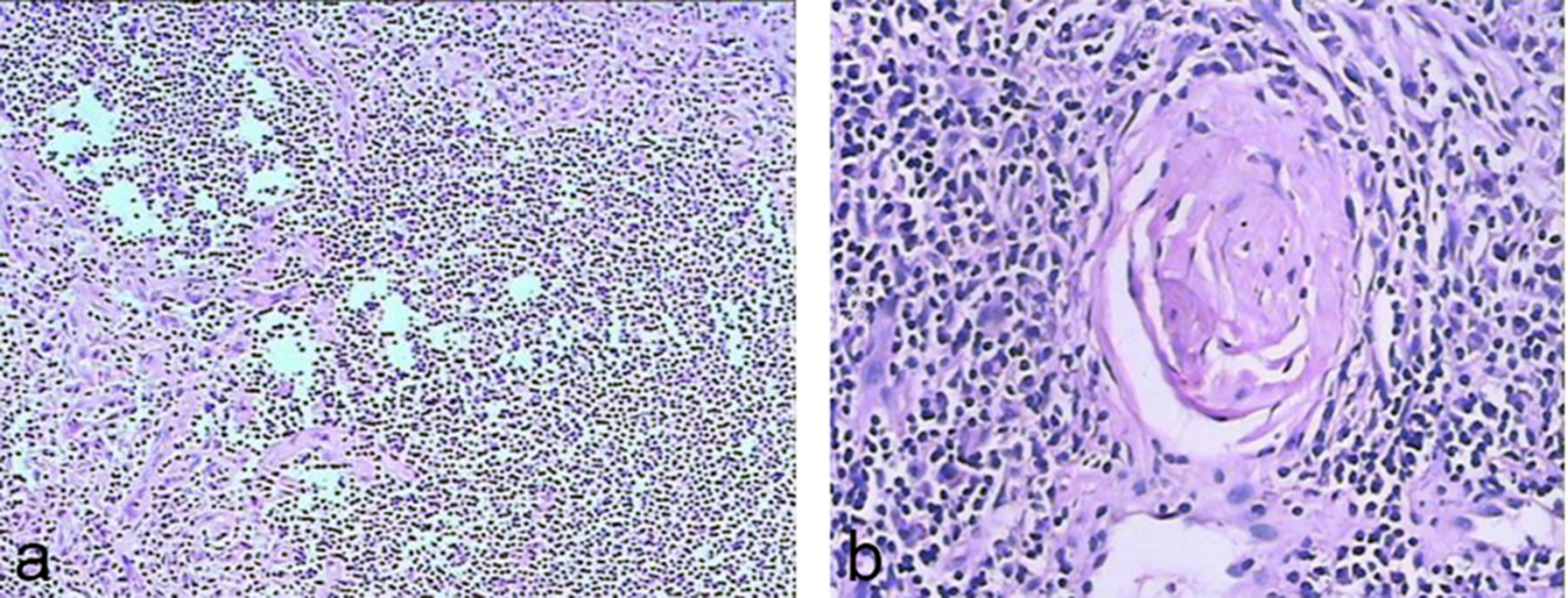
Microscopic appearance of the lesion. (a, b) Haematoxylin and eosin staining (magnification 10 × 10, 10 × 20). (a) Hyperplasia of blood vessels, lymphoid tissue and lymphoid follicles in the lesion. (b) Germinal centre of the follicle.

A 43-year-old female with dyspnoea was transferred to our emergency department for further treatment. She had dyspnoea and cough about 6 months ago. No sputum or haemoptysis was observed. She worked as a farmer for about 10 years, and such symptoms had never happened. Occupational poison contact was denied. Her parents were healthy, with no similar family history or medical history. Empiric anti-infective therapy and antituberculosis therapy were given in the last few months at a local hospital, but the symptoms of dyspnoea worsened. Plain CT of the chest revealed a mass with well-circumscribed soft-tissue attenuation, which measured about 25 Hounsfield units (HU) on the lateral wall of the trachea (Figure 1a,b). It was about 2 × 1 × 1 cm^3 ^in size. Contrast-enhanced CT revealed marked homogeneous enhancement (Figure 2a–c), with the highest density of 122 HU in the arteries that declined gradually to 85 HU at 120 s after injecting the contrast medium. No metastatic lymph nodes or infiltration into nearby structures was observed. No similar lesions were found in the lungs, mediastinum or abdomen. Tracheotomy was arranged. Macroscopically, the mass was about 2 × 1 × 1 cm^3^ with a distinct border and smooth surface. No enriched vasculature was observed. Microscopically, the lesion was composed of a large amount of lymphatic tissue and hyperplastic vascular and lymphoid follicles, with atrophy of the germinal centre (Figure 3a,b). Immunohistochemical staining of the specimen confirmed the diagnosis of Castleman’s disease (CD). The specimen was CD20(+)/CD79(+) in B lymphocytes and CD3(+)/CD5(+) in a few T lymphocytes. Plain CT of the chest 3 months after the surgery excluded relapse or residual lesions in the trachea. The patient was then under regular follow-up, and her physical condition was quite good.

## Discussion

CD, which was first described in 1954 by Benjamin Castleman, is an uncommon lymphoproliferative disorder of uncertain causes.^1,2^ It can occur at any site with lymphatic tissue throughout the body. Of all lesions reported, 70% were in the chest, 15% were in the neck and the remaining were in the abdomen and pelvis.^3^ CD may be asymptomatic and discovered incidentally. It can present with painless lymphadenopathy or other symptoms related to structures compressed nearby.^3^ CD in trachea is rarely reported. Like other lesions in the trachea, obstruction of the airway and progressive dyspnoea may be the main symptoms, but it is far from diagnostic.

As tracheal lesions have an overall low prevalence, it is easy for radiologists and doctors not to pay enough attention to them, thus making tracheal lesions “forgotten zones” in chest imaging,^4^ even for chest radiologists. Preoperative diagnosis of such focal lesions in trachea remains challenging for several reasons. On one hand, various lesions including inflammatory diseases, neoplastic lesions and systemic lesions can occur.^5^ Squamous cell carcinoma and adenoid adenoma have the highest occurrence rate.^5^ Squamous cell papilloma, non-Hodgkin’s lymphoma and papillomatosis should be taken into consideration in differential diagnosis, as they can occasionally be seen in clinical practice.^5^ On the other hand, even though some focal tracheal lesions have very low morbidity and are reported in case reports, they can share similar clinical and radiological presentations with CD. These focal hypervascular lesions may include paraganglioma and haemangioma. Besides, another reason that makes accurate preoperative diagnosis challenging is that CD may have various presentations according to its pathological type.

On imaging, the classic CT finding of hyaline vascular-type CD is a solitary enlarged lymph node or localized nodal mass that demonstrates intense homogeneous enhancement. Approximately 10% of the lesions have internal calcifications, with characteristic coarse or distinctive branching patterns.^6^ On MRI, they usually exhibit as heterogeneous *T*_1_ and *T*_2_ hyperintensity signals compared with the skeletal muscle.^7^ Prominent flow voids, which imply the existence of feeding vessels, may be seen. On positron emission tomography-CT imaging, the lesions can show a high uptake of radiolabelled [^18^F]-2-fludeoxy-D-glucose, thus mimicking malignant adenopathy.^8^ Ultrasound plays an important role in detecting superficial lesions, which usually exhibit as single, well-defined, hypoechoic solid masses with no cystic necrosis inside.^6^

CD originating from the trachea is rare. Only two cases^9,10^ have been reported in English literature, both of them presented with respiratory distress and both lesions were resected with rigid bronchoscopy.^10^ Tracheotomy remains a classic treatment for similar lesions in trachea, and bronchoscopy is another solution widely used in recent years. Rigid bronchoscopy is a less invasive method, while tracheotomy enables more complete excision of lesions, especially for potentially malignant cases. In this patient, malignant lesions were suspected before surgery and tracheotomy was arranged to remove the lesion.

## Learning points

1. CD can occur at any site with lymphatic tissue; a mass or a group of masses with a distinct margin and an obvious enhancement on CT might be an implication of CD.
2. A suggestive preoperative diagnosis of lesions in trachea can help doctors make more appropriate operative plans.

## Consent

The patient agreed the doctors could use and publish her disease related article with personal information deleted.
